# Suppression of Parathyroid Hormone in a Patient with Severe Magnesium Depletion

**DOI:** 10.1155/2016/2608538

**Published:** 2016-04-14

**Authors:** Sangeeta Mutnuri, Isaac Fernandez, Tina Kochar

**Affiliations:** Department of Nephrology and Hypertension, University of Texas Medical Branch, 4.200 John Sealy Annex, Galveston, TX 77555-0562, USA

## Abstract

Hypomagnesemia is often associated with coexisting electrolyte abnormalities like hypokalemia and hypocalcemia. Hypocalcemia has been shown to be secondary to hypoparathyroidism induced by hypomagnesemia. Here, we discuss a case of a patient with severe hypomagnesemia and associated hypocalcemia. A 38-year-old lady was admitted to the hospital for weakness of lower extremities and an eventual fall. The exam was significant for decreased motor strength and some paresthesias. The laboratory data was significant for hypomagnesemia, hypokalemia, and low parathyroid level in the face of hypocalcemia. After replacing magnesium, the parathyroid hormone levels normalized and led to eventual correction of calcium levels without any additional calcium replacement therapy. There was complete symptom resolution with correction of electrolyte abnormalities. This case highlights the importance of looking for all associated abnormalities in a patient with hypomagnesemia and starting the replacement therapy by first replacing the magnesium and then the others as needed. Replacing the magnesium alone may correct the hypoparathyroidism and eliminate the need for calcium replacement.

## 1. Introduction

Hypomagnesemia is a common electrolyte abnormality found in about 12% of hospitalized patients [1]. The incidence rises to as high as 30% in patients with a history of chronic alcohol intoxication [2]. Deficient magnesium levels are largely due to poor nutritional intake and increased renal and gastrointestinal losses of magnesium, all of which are markedly present in alcoholic patients. As is the case with the patient reported, symptomatic magnesium depletion is often associated with multiple biochemical abnormalities such as hypocalcemia and hypokalemia. Common manifestations of the electrolyte imbalances include neuromuscular irritability such as weakness, cramps, paresthesias, fatigue, dysgeusia, and anorexia.

We report the case of a patient presenting with hypocalcemia and hypokalemia and inappropriately normal parathyroid hormone levels complicating severe magnesium depletion. Our observation illustrates that magnesium deficiency can induce hypoparathyroidism and therefore subsequently lead to hypocalcemia.

## 2. Case Presentation

A previously healthy, 38-year-old lady with a history of chronic alcohol use (0.5–1.0 gallon of vodka per day) presented with a three-week history of progressive weakness and generalized fatigue. She complained of paresthesias and weakness in her feet, which progressed proximally and involved her hands. She presented to the hospital after falling and injuring her feet due to inability to walk. She had previously seen her primary care physician and was started on folate supplements. She also complained of diarrhea but denied any anorexia, dysgeusia, cramps, or spasms. On physical examination, the patient was noted to be afebrile, and her blood pressure was 148/78 mmHg. Cardiopulmonary and abdominal exam were normal. Neurological exam showed intact cranial nerves, 4/5 strength bilaterally in upper and lower extremities, diminished triceps, and patellar and Achilles tendon reflexes. There was decreased sensation to temperature, pinprick, vibration, and proprioception that followed a stocking/glove distribution up to the knees and elbows, bilaterally. Neither Chvostek's nor Trousseau's signs were present. Laboratory analysis revealed the following: potassium: 2.9 mEq/L (3.5–5.0), total calcium: 7.3 mg/dL (8.6–10.6), albumin: 3.4 g/dL (3.0–4.8), magnesium: 0.8 mg/dL (1.7–2.4), phosphorus: 5.0 mg/dL (2.5–5.0), intact parathyroid hormone (iPTH): 31.8 pg/mL (12–88), 25 (OH) vitamin D3: 9 ng/mL (25–80), creatinine: 0.66 mg/dL (0.5–1.04), bicarbonate: 25 mmol/L (23–31), AST: 162 U/L (13–40), ALT: 61 U/L (9–51), lipase: 471 U/L (0–220), folate: 5.6 ng/mL (3–20), vitamin B12: 442 pg/mL (240–930), tissue transglutaminase IgA: 3 units (0–19), and hemoglobin: 11.8 g/dL (11.6–15.0). In addition, the patient was noted to have elevated homocysteine levels and normal levels of B12 and folate. Urine studies were significant for urine potassium: 27.7 mmol/L, urine calcium: 20 mg/dL, urine magnesium: 23 mg/dL, and urine creatinine: 103.6 mg/dL. The fractional excretion of magnesium was calculated to be 26%, indicating renal magnesium wasting. The significant electrolyte abnormalities as well as possible folate and B12 deficiency seemed to account for her generalized weakness. Her history of heavy alcohol consumption was thought to have caused her laboratory abnormalities and symptoms. The patient was treated initially with two grams of magnesium intravenously for one day and then switched over to oral magnesium oxide replacement (800 mg three times a day) for seven days. She was also given vitamin B12 and folate replacements. Given the vitamin D deficiency on the labs, which could contribute to the hypocalcemia, the patient was discharged on high dose of vitamin D supplementation.

**Figure 1:**
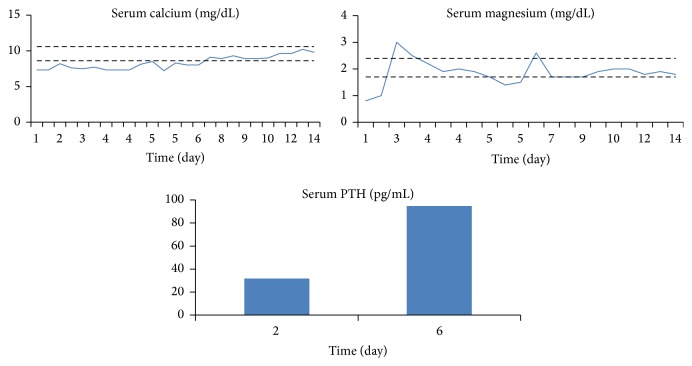
Levels of serum calcium and magnesium under magnesium supplementation (normal values are represented in dashed lines).

The normal iPTH levels in the setting of hypocalcemia seemed inappropriate. Once the hypomagnesemia was corrected, the iPTH levels were rechecked and an appropriate rise was noted. Soon after, the hypocalcemia resolved as well. The preceding treatment allowed for an appropriate rise in her PTH levels, which then allowed for the correction of the hypokalemia and hypocalcemia that had been refractory to oral replacements (Figure 1). The patient's overall condition and weakness improved after two weeks of treatment.

## 3. Discussion

There is no hormonal control in place for magnesium balance within the body. Due to its concomitant presentation with other electrolyte abnormalities, magnesium deficiency can present in various ways. The most common signs of magnesium deficiency are hypokalemia and hypocalcemia [1]. Patients with symptomatic hypomagnesemia will most commonly present with neuromuscular irritability, as was the case with our patient. The case presented illustrates a situation in which a patient presented with severe biochemical disorders secondary to chronic alcohol consumption combined with acquired suppression of iPTH in the face of hypocalcemia. The suppression of iPTH resulted from severely decreased total-body magnesium as evidenced by the rise in iPTH levels after replenishing the magnesium stores.

Chronic alcoholism is a well-known cause of magnesium deficiency [2]. Within this subset of patients, some of the mechanisms involved are poor nutritional intake, increased gastrointestinal losses in vomiting and diarrhea, decreased absorption secondary to pancreatic insufficiency or vitamin D deficiency, and alcohol induced tubular dysfunction which leads to increased magnesium urinary excretion [1, 3, 4]. De Marchi et al. discuss various electrolyte abnormalities including hypomagnesemia, which occur as a result of renal tubular defects in patients with history of heavy alcohol use. The values normalized after 4 weeks of alcohol abstinence indicating a direct correlation. Interestingly, the patients in the study were noted to have low parathyroid hormone levels, as seen in our patient [5].

Hypomagnesemia is associated with a reduction in intracellular magnesium concentration, which may then lead to a decline in adenosine triphosphate (ATP) activity. This decline removes the ATP inhibition of potassium channels, leading to an increase in the number of these channels [6]. Moreover, a decrease in intracellular magnesium has been shown to directly increase the activity of potassium channels of the ascending limb cells [7]. As a result, the hypokalemia in this setting is relatively refractory to potassium supplementation and requires correction of the magnesium deficiency.

Several possible mechanisms have been identified to explain the hypocalcemia seen in patients with deficient magnesium levels. In addition to poor nutritional intake, in many magnesium depleted patients iPTH concentrations are either inappropriately normal, as in our case, or low, suggesting inhibition of iPTH secretion or synthesis [4, 8–10]. On the other hand, magnesium depleted patients who present with hypocalcemia despite high iPTH levels suggest bone and kidney resistance to parathyroid hormone [11, 12]. Hypocalcemia secondary to peripheral PTH resistance or decreased PTH secretion, as in our case, is further complicated by the loss of PTH stimulation of 1-alpha-hydroxylation of vitamin D in the kidney, thereby worsening the vitamin D deficiency [13].

Magnesium plays a central role in adenylate cyclase activity and subsequently in the production of cyclic adenosine monophosphate (cAMP) [14, 15]. Since both iPTH secretion and end organ effects of iPTH are mediated by cAMP, altered adenylate cyclase function in magnesium deficiency causes impairment in iPTH levels [4, 14, 15].

In conclusion, the case presented demonstrates that magnesium therapy alone is able to help correct hypocalcemia secondary to iPTH suppression. This observation emphasizes the importance of checking magnesium levels in a patient presenting with hypocalcemia and the importance of therapy with magnesium in these biochemical abnormalities. Depending on the degree of magnesium deficiency and the clinical symptoms, aggressive intravenous therapy may be necessary. An abrupt elevation in serum magnesium concentration will partially remove the stimulus for magnesium retention, and up to 50% of the infused magnesium will be excreted in the urine [1]. Therefore, magnesium repletion requires sustained correction in the form of oral replacement in order to avoid recurrence of hypomagnesemia.
